# Multiple-batch spawning: a risk-spreading strategy disarmed by highly intensive size-selective fishing rate

**DOI:** 10.1098/rspb.2022.1172

**Published:** 2022-08-31

**Authors:** Sara Hočevar, Jeffrey A. Hutchings, Anna Kuparinen

**Affiliations:** ^1^ Department of Biological and Environmental Science, University of Jyväskylä, Jyväskylä 40014, Finland; ^2^ Department of Biology, Dalhousie University, Halifax NS B3H 4R2, Canada; ^3^ Institute of Marine Research, Flødevigen Marine Research Station, N-4817 His, Norway; ^4^ Department of Natural Sciences, University of Agder, N-4604 Kristiansand, Norway

**Keywords:** Atlantic cod, bet-hedging, fitness, fisheries-induced evolution, multiple-batch spawning, size-selective fishing

## Abstract

Can the advantage of risk-managing life-history strategies become a disadvantage under human-induced evolution? Organisms have adapted to the variability and uncertainty of environmental conditions with a vast diversity of life-history strategies. One such evolved strategy is multiple-batch spawning, a spawning strategy common to long-lived fishes that ‘hedge their bets' by distributing the risk to their offspring on a temporal and spatial scale. The fitness benefits of this spawning strategy increase with female body size, the very trait that size-selective fishing targets. By applying an empirically and theoretically motivated eco-evolutionary mechanistic model that was parameterized for Atlantic cod (*Gadus morhua*), we explored how fishing intensity may alter the life-history traits and fitness of fishes that are multiple-batch spawners. Our main findings are twofold; first, the risk-spreading strategy of multiple-batch spawning is not effective against fisheries selection, because the fisheries selection favours smaller fish with a lower risk-spreading effect; and second, the ecological recovery in population size does not secure evolutionary recovery in the population size structure. The beneficial risk-spreading mechanism of the batch spawning strategy highlights the importance of recovery in the size structure of overfished stocks, from which a full recovery in the population size can follow.

## 1. Introduction

More than 34 000 species of fish have been described so far [1]. The diversity of life histories among them is vast and even exceeds the number of species [2]. Each species developed a unique strategy to allocate its energy budget among the selected life-history traits, each one with the ultimate goal: to maximize the number of next-generation spawners, fitness, before being vanquished by natural mortality [3,4].

Some species have adapted to minimize the high natural mortality rates and increase the survival of the earliest life stages with reproductive strategies in spawning patterns [5]. Some teleosts optimize their reproductive success and reduce the risk of offspring mortality with spawning location and are demersal spawners—spawning their eggs on different kinds of a substrate (e.g. capelin *Mallotus villosus* [6]), while some are pelagic spawners—spawning their eggs into a water column (e.g. European pilchard *Sardina pilchardus* [7]). Extensive variability also exists in spawning regularity and the number of spawning episodes. Species may be semelparous spawners, spawning all the eggs only once in their life (e.g. Chinook salmon *Oncorhynchus tshawytscha* [8]), or iteroparous spawners, spawning eggs for multiple years. The former can be parted into total spawners, which spawn a batch of eggs once within a season (e.g. Atlantic herring *Clupea harengus*) or multiple-batch spawners (MBS), which spawn several batches of eggs within a season (e.g. haddock *Melanogrammus aeglefinus*). Some batch spawning fish may even skip spawning in years when environmental cues favour investment into somatic growth instead of reproduction [9]. Batch spawners can be further classified as determinant batch spawners (e.g. European sea bass *Dicentrarchus labrax* and plaice *Pleuronectes platessa*) or indeterminate batch spawners (e.g. European anchovy *Engraulis encrasicolu*s and sole *Solea solea*), depending on whether the number of vitellogenic oocytes is determined before the start of the spawning season or whether it is formed continuously throughout the spawning season [10,11].

Spawning strategies of teleosts, characterized by the spawning frequency and fecundity have been fine-tuned over hundreds of millions of years of evolution to the optimal trade-off among survival, growth and reproductive traits to challenge natural mortality. But how do these evolutionary winning strategies perform when exposed to the *non-natural mortality* of the modern epoch? Could the advantage of risk-mitigating spawning strategies become a disadvantage under human-induced mortality?

Reconstructions from historical records have demonstrated that some of today's commercially fished stocks represent merely a remnant of once highly abundant populations [12,13]. For example, cod fisheries date back to the pre-Mesolithic Stone Age [14], but intense fishing practices have depleted most stocks only in recent decades, with some stocks collapsing to less than 5% of their pre-industrial fishing values [13]. As a consequence, some stocks may have passed their tipping point of recovery as reflected by the Allee effects [15,16]. Regardless of stricter fisheries regulations, moratoriums and the decades passed, stocks such as Canadian stocks, have yet to recover their biomasses from their critical zones [13].

In the present study, we explore the extent of the impact that size-selective fishing has on the risk-spreading strategy of MBS. Our focal species is the iconic Atlantic cod (*Gadus morhua*), a determinant demersal spawner with a wide geographic range and a long history of fishing exploitation [17]. Life-history characteristics set cod among the most fecund species with a long reproductive lifespan, late maturation, increasing maternal investment with age and repetitive broadcast spawning. Some of these reproductive traits, including the duration of the spawning period and the frequency of spawning events, tend to correlate positively with body size in cod [18,19]. The spawning dynamics of cod, in which fish shed eggs in multiple batches within every spawning season [18], grant a spatial and temporal risk-spreading effect [20]. This diverse distribution of egg batches contributes to higher across-generational fitness and can act as a bet-hedging strategy under environmental conditions when odds of survival are low and hard to predict [21].

We apply an individual-based eco-evolutionary model [22] to investigate the performance of batch spawning strategy under human-induced mortality, in form of size-selective fishing. To encompass the wide spectrum of risk that this risk-spreading strategy provides, from risk-averse, where eggs are shed across as many batches as the female's body size can support, to risk-prone, where all the eggs are shed at once, we compare multiple-batch spawning cod to its hypothetical opposite: a single-batch spawning cod. With this approach, we disentangle the far-reaching effects that the batch spawning strategy as such promotes. In particular, we look into the influence of spawning strategy on (i) fisheries-induced evolution of fish body size, (ii) changes in fitness dynamics and survival of recruits that join the adult population, and (iii) alteration of population age structure. Overall, we highlight the eco-evolutionary role of a multiple-batch spawning strategy under size-selective fishing and show that this risk-spreading strategy is not resilient to fishing selection.

## 2. Methods

### (a) Extended mechanistic cod model

The individual-based mechanistic model, developed and thoroughly described by Kuparinen *et al*. [22], follows each individual cod within a population at each annual time step from the start to the end of its simulated life. In the years between, the individuals undergo the processes of natural and fishing mortality, growth, and, if mature, reproduction. The fish lengths (*L*) follow their von Bertalanffy growth trajectories [23], and the integral evolving trait of the model is the asymptotic maximum length *L_∞_.* This model parameter represents the basis for other size-related life-history [24] and fitness traits [18,19]. To create an initial genetic pool of *L_∞_* values, the model integrates the negative correlation between the growth rate *k* and *L_∞_* estimated from 258 growth trajectories of an unexploited cod population from a lake in the Canadian Arctic [25].

At the time of birth, each recruit inherits a unique genetic value of *L_∞_* from its parents via classical Mendelian principles. After accounting for normally distributed environmental noise (s.d. = 3.5) around the genotypic value (ranging between 0 and 20), the phenotypic value of *L_∞_* is set, resulting in a naturally observed heritability of 0.2–0.3 [26]. The ratio between the population's biomass and carrying capacity in the corresponding year was used as a measure of density dependence, and it negatively affected the somatic growth of individuals along their von Bertalanffy growth curves [22].

### (b) Spawning strategy

The spawning season occurs once a year for every individual that reaches the maturation threshold set to 66% of its individual asymptotic maximum length *L_∞_* [27]. The number of eggs a mature female produces is positively correlated to its body weight (table 1), following empirically based age-specific fecundity [28], while the weight is derived from the empirically based length–weight relationship [22,28].

**Table 1.** Descriptions of equations and sources underpinning the empirically derived variables of the individual-based mechanistic model.

description	equation	source
length (*L*)–weight (*W*) relationship	$W_{t\;}=3.52\cdot 10^{-6}\cdot L_{t}^{3.19}$	Kuparinen *et al*. [22]
age-specific fecundity (*N*_eggs_)	$N_{\mathrm{eggs}}=\;\left(\frac{0.48\cdot (W_{t}+0.37)}{1.45}+0.12\right)\cdot 10^{-6}$	Hutchings [28]
batch number (*N*_batches_)	$N_{\mathrm{batches}}=\frac{21.156}{1+\exp((55.014-L_{\mathrm{fork}(t)})/10.141)}$	derived in Hočevar *et al*. [21] based on empirical data from Roney *et al*. [29]
multiple-batch spawning costs (*Costs_MBS_*)	${Costs}_{MBS}=\;\overset{N_{\mathrm{batches}}}{\underset{\mathrm{batch}=1}{\sum }}1-0.00523\;\cdot (\mathrm{batch}-1)$	derived in Hočevar *et al*. [21] based on empirical data from Roney *et al*. [29]
size-based fishing *selectivity* (*L(t)* is the length of an individual at the time of fishing)	$selectivity=\frac{\exp(-12.5+0.25\;\cdot \;L(t))}{1+\exp(-12.5+0.25\cdot L(t))}$	Kuparinen & Hutchings [30]

To identify the impact of size-selective fishing on spawning dynamics, we simulated populations in the presence and absence of a multiple-batch spawning strategy. This allowed us to separately generate and track the populational dynamics of multiple-batch and single-batch spawning individuals. Each batch spawning female (i.e. a *MBS*) imitates the risk-spreading strategy of Atlantic cod. Such a female spawns its eggs in multiple batches in several pulses during an extended spawning season. The number of spawned batches is positively correlated to female body size [19] and the relation is derived from the fitted data on the number of batches and female fork length *L*_fork(*t*)_ (table 1) gathered on cod from coastal Skagerrak (fig. 2 in [21]). We implemented batch spawning costs to the model because we consider multiple-batch spawning a bet-hedging trait [21] which acts with associated costs to mean arithmetic fitness [31–33]. Spawning mortality costs gradually increase from 0 to 0.11 for every subsequently shed egg batch, to reflect the empirical observation of Norwegian coastal cod [19] (fig. 2 in [21]), where larval length and yolk-sac volume declined in the latest batches. We applied the spawning costs separately as a decreased survival probability to each shed egg batch. On the other hand, the model assumes that every female that is not a MBS, but a *total* or *single-batch spawner* (SBS), releases all eggs annually in a single spawning event and suffers no associated spawning costs.

### (c) Environmental forcing

We introduced the environmentally induced increase in mortality rate, hereafter environmental forcing, into the model through a batch mortality rate. We tested six rates of environmental forcing (0.05, 0.10, 0.15, 0.20, 0.25 and 0.30). The interval captured the most sustainably endurable environmental mortality (higher tested rates resulted in frequent collapses among populations). To simplify the study design, we considered two contrasting scenarios where an entire egg batch dies or survives. The outcome is uniquely drawn randomly each time for each shed batch, imitating the risk-spreading effect of shedding multiple batches. The environmental forcing rates are applied to each batch individually along with the related spawning costs as a success probability in a Bernoulli trial every time a MBS produces an egg batch. Similarly, the environmental forcing is introduced to SBS in such a way that the survival of a single produced batch is drawn randomly based on a Bernoulli trial and determines whether the batch survives or dies.

### (d) Recruitment

The final number of individuals that a MBS or a SBS recruits to the population is determined by applying the juvenile survival probability, as the probability of survival from egg to recruit, 1.13 × 10^−6^ [28] in a Bernoulli trial to the sum of all eggs of every batch for the first 3 years of individual's life. Once the offspring reaches 3 years of age, they experience an annual instantaneous rate of natural mortality (*M*) of 0.15, which at maturity increases by 0.1 due to reproductive costs. The lifespan maximum of every individual cannot exceed 25 years [22].

### (e) Size-selective fishing

During the fishing season, every individual that is longer than the minimum size threshold of 45 cm can be fished, following the empirically parameterized sigmoid size-selective model (table 1). The model imitates the most used fishing gear in cod fisheries—the bottom trawls—which tend to exert stronger selection on larger sized individuals (electronic supplementary material, figure S1). We applied the product of a pre-determined instantaneous fishing mortality rate, *F* (year^−1^), (*F* = 0.1, 0.2 and 0.3) and an individual-based selectivity as the survival probability in a Bernoulli trial [30] to determine whether the individual is captured by the trawl or not. We discontinued trawling as soon as the population biomass dropped to 15% of its initial pre-fishing capacity.

### (f) Simulation design and analyses

**Figure 1.**
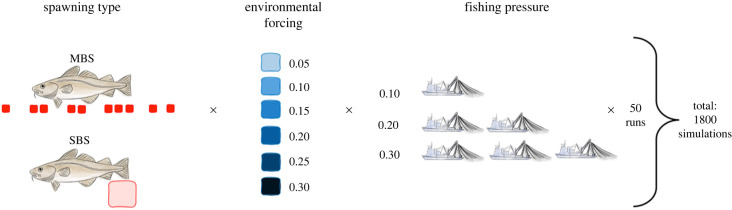
Schematic demonstration of the study's simulation design. Fish populations consisted of individuals with one spawning type: MBS or SBS. While fish with the former strategy shed their eggs among multiple batches within each spawning season, the fish of the later strategy deployed the same number of eggs in one spawning event. We performed 50 replica simulations of every population exposed to one of six environmental forcing rates and one of three fishing pressure rates, linked in a multi-factorial manner.

We investigated the response of spawning dynamics to fishing pressure by running the model under separate scenarios and analysing their outputs. As a result, 50 replicates for each of the 36 scenarios were simulated (figure 1). Each population, representing one of the two spawning strategies, was exposed to six environmental forcing rates (0.05, 0.10, 0.15, 0.20, 0.25 and 0.30) and three fishing mortality rates (0.1, 0.2 and 0.3) in a full factorial manner. Instantaneous fishing mortalities greater than 0.30 resulted in increasingly rapid population depletions, limiting our resolution of the ongoing populational dynamics during fishing, and were thus not investigated.

We allowed the populations, of which the initial size was 2000 individuals, to adapt to the simulated environmental forcing scenarios for 5000 years before starting the *in silico* experiment. Then, each of the pre-adapted 36 scenarios was simulated for 500 years, wherein the first 100 years, the populations lived under the corresponding environmental forcing, in the following 300 years (or until the biomass dropped to 15% of its initial condition) under environmental forcing and added fishing pressure, and in the last 100 years (or the remaining years until year 500) again solely under environmental forcing. During each simulated time step, we recorded population biomass and abundance, catch biomass and individuals' asymptotic maximum length *L_∞_*, maturation age and size. We also followed the across-generational fitness, derived as the geometric mean or the *n*th root of the product of average realized lifetime reproductive generational output [33].

Simulations and data analyses were conducted in the open-source statistical programming language R [34]. We used a collection of R packages ‘tidyverse’ for data visualization [35].

## 3. Results

### (a) Evolution of life-history traits under natural and fishing selection

**Figure 2.**
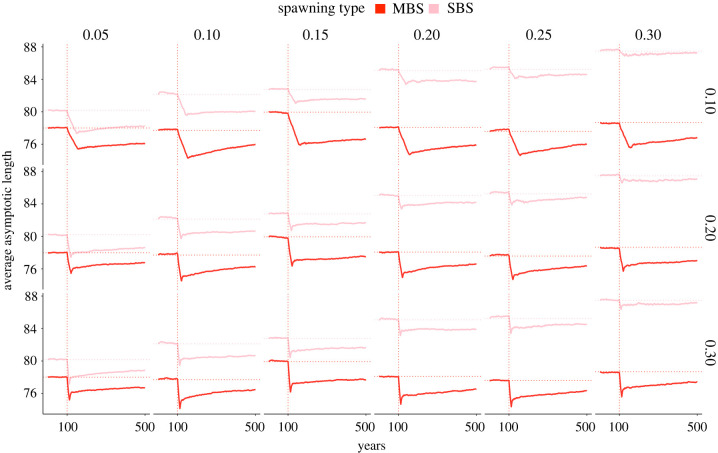
Selection in asymptotic length (*L_∞_*) of Atlantic cod under environmental forcing rates and fisheries-induced pressure. The average asymptotic lengths of the populations with MBS (red coloured line) and SBS (light pink coloured line) strategy are plotted against time. The panels indicate row-wise the fishing effort and column-wise the environmental forcing, applied to the populations throughout the entire simulation. The vertical dashed line illustrates the onset of fishing that was ceased as soon as the population dropped to 15% of its initial biomass and horizontal dashed lines depict the value of *L_∞_* before the fishing started.

The evolution of *L_∞_* distinctively differed between the two spawning strategies (figure 2). In both cases, *L_∞_* began declining immediately after the start of size-selective fishing, illustrating the selective direction of fishing pressure towards smaller body sizes. The proportional reduction in *L_∞_* differed between the multiple-batch and single-batch spawning strategists. A population comprised MBS exhibited more substantial fisheries-induced selection towards smaller body sizes, which dropped in general by approximately 3% and had a steeper negative slope with increasing realized fishing mortality rate, compared to a population comprised SBS. This led to MBS being on average 1.36 cm smaller than SBS under the same fishing mortality rates.

Single-batch spawning populations showed a tendency in selection for bigger body size as the environmental perturbations increased while *L_∞_* of MBS showed no such trend and remained within a narrow range despite changes in environmental forcing. Fishing ceased under all simulated scenarios when populations declined to 15% of the initial biomass. Shortly after the cessation of fishing, the declining trend in *L_∞_* of both spawning strategies ceased and thereafter began a slow recovery process, which was not fully reached even after more than 300 years long moratorium or no-fishing period. Although SBS exhibited stronger recovery potential in *L_∞_* than MBS (figure 2), they also exhibited higher sensitivity to environmentally induced perturbations, evidenced by a prolonged lag in its post-fishing recovery in biomass to carrying capacity (electronic supplementary material, figure S2). The greater the environmental perturbations, the longer the lag time to rebuild the pre-fishing biomass. The multiple-batch spawning population showed no such sensitivity to environmental perturbations.

### (b) Fitness shaped by fishing

**Figure 3.**
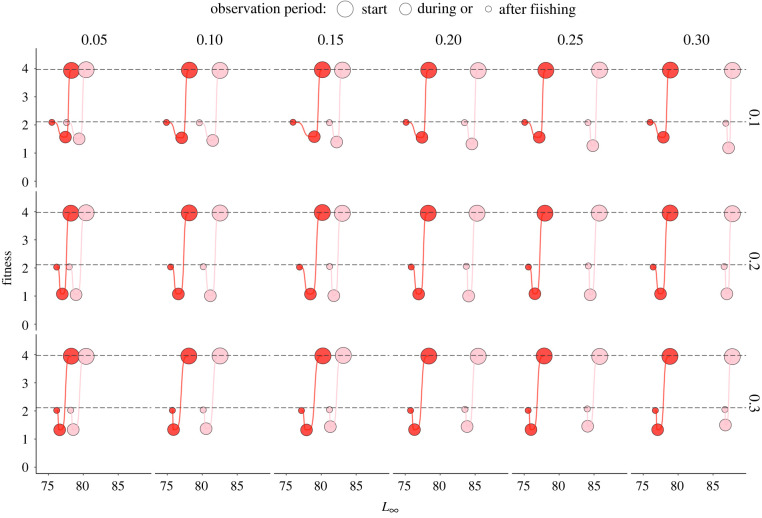
Fitness before, during and after fishing period calculated as a geometric mean of realized reproductive output across cohorts living in that period. Fitness is plotted against average asymptotic length (*L_∞_*) and faceted according to fishing pressure (row-wise) and environmental forcing (column-wise). The red and pink points represent the mean values of 50 replica simulations for each scenario combination of multiple (MBS) and single (SBS) batch spawning strategists, respectively. The black dashed lines indicate the highest fitness value before (3.97) and after (2.10) fishing period.

The type of spawning strategy and exposure to environmental forcing and fishing pressure influenced the across-generational fitness (figure 3). The fitness of both strategists declined by up to 75% during intense harvest and recovered after fishing was ceased to 53% of the pre-fishing value (for a closer view of initial fitness: electronic supplementary material, figure S3A). The recovery rate was the lowest under the strongest fishing pressure (recovered to 51% of pre-fishing value).

Multiple-batch spawning populations had higher fitness than single-batch spawning populations during fishing when fishing intensity was 0.10 and 0.20, and lower fitness when fishing intensity was 0.30 (for a closer view: electronic supplementary material, figure S3B). Differences in fitness between the two strategies increased with increasing environmental forcing, as multiple-batch spawning populations were not sensitive to environmental change, while single-batch spawning populations were. Post-fishing fitness of multiple-batch spawning populations was higher than that of the single-batch spawning populations only after recovering from the least intensive fishing period (0.10), while single-batch spawning population showed better recovery in fitness after more intensive fishing periods (0.20, 0.30) (for a closer view: electronic supplementary material, figure S3C). Overall, compared to populations comprised SBS, multiple-batch spawning populations experienced a lower proportion of failed spawning seasons before, during and after fishing. The proportion of failed events among MBS increased from approximately 0.4 before fishing to approximately 0.6 during fishing and did not change with increasing environmental mortality, while fishing mortality had an effect (electronic supplementary material, figure S4).

We did not observe any consistent trend in the average realized individual fitness, calculated as the abundance of recruits divided by the abundance of mature adults, except that the post-fishing realized individual fitness of MBS was always lower than before and during fishing (maximum difference for MBS: 0.026 and for SBS: 0.015) (electronic supplementary material, figure S5). We also found that the interannual variance in individual fitness increased with fishing intensity during fishing and was lower among MBS (electronic supplementary material, figure S6).

### (c) Length of fishing period and catch biomass

Recovery in recruitment abundance began once population biomass dropped below 15% of initial biomass and fishing activities ceased. Under the lowest fishing pressure, the multiple-batch spawning strategy enabled the population to endure size-selective fishing for 9 to 46 years longer than SBS (median length of the fishing period at 0.10 for MBS 51 years and for SBS 35 years). However, under fishing pressure of 0.20 and 0.30, the resilience of the multiple-batch strategy to fishing mortality decreased or even vanished, reducing the difference between MBS and SBS to 2–8 years and 0–4 years, respectively (percentage comparison in the electronic supplementary material, figure S7).

Multiple-batch spawning strategy kept the biomass of recruits consistently higher compared to SBS, and the difference between the two strategies in their recruitment abundance increased with increased environmental forcing, from the lowest difference of 8% under the least fatal environment to the biggest difference of 36% under most fatal environment (electronic supplementary material, figure S7). The advantage under most fatal environmental conditions decreased with greater fishing pressure, to 35% and 32% under 0.20 and 0.30 of fishing pressure, respectively, and consequently, fishing had to be terminated sooner.

Catch biomass decreased with increasing environmental mortality rates, and the decrease was steeper among SBS compared to MBS. By contrast, increasing fishing pressure resulted in higher annual catch biomass, with its trend steeper and higher among multiple-batch than single-batch spawning cod. The total catch biomass of both strategists decreased with higher fishing intensity (electronic supplementary material, figure S8).

### (d) Age structure

**Figure 4.**
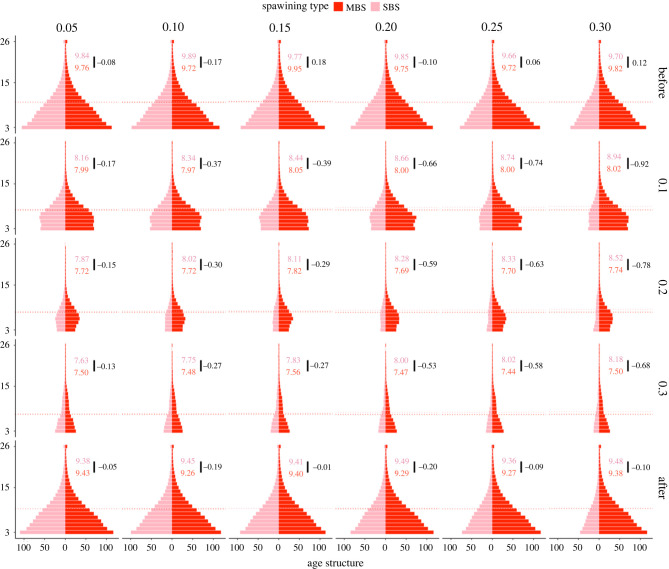
The age structure of a multiple (MBS; red coloured) and single (SBS; pink coloured) batch spawning cod population. Row-wise the panels depict the demographical structure of cod stock before fishing, during increasing fishing pressure and after fishing. The changes in the populational age structure can be observed also among environmental forcing rates (column-wise). The horizontal dashed lines indicate the average age of each population pyramid and numbers show the difference in the average age at maturation between the strategies.

To determine whether spawning strategy affects the overall age distribution of cod stocks, we portrayed the pre-fishing, during-fishing and post-fishing cohort abundance in populational pyramids of corresponding years (figure 4). In the pre-fishing period, younger classes dominated both strategies, reflecting a fast-growing pace of populations. Introduced fishing changed the distribution of age classes of cod populations considerably by shifting the shape of the population pyramid from expansive towards constrictive. In particular, under the fishing mortalities of 0.1 and 0.2, the populational pyramid narrowed at the bottom, illustrating a decreasing influx of recruits. Younger classes reclaimed the dominance under the highest fishing intensity 0.3, but the shape was not as triangular as in the pre-fishing time. In terms of abundance, the differences between the two strategies became more pronounced with increased environmental forcing, as the multiple-batch spawning strategy showed higher reproductive potential.

During the rebuilding phase, both strategies recovered their shape of populational age structure to that of the pre-fishing status, but SBS did not manage to recover to the pre-fishing abundance. The post-fishing populational pyramids remained slimmer under scenarios of greater environmental forcing. MBS had an overall higher mean age compared to SBS (13.44 years and 13.25 years, respectively), but as the fishing intensity increased, the difference between the two strategies decreased with an advantage accruing to SBS, who had higher mean age under the greatest fishing pressure (0.3), showing lower sensitivity to fishing than MBS as their average age decreased more rapidly and intensively (MBS: 26% or 14.22–10.66 and SBS 20% or 14.03–11.23). On the other hand, the mean populational age of MBS remained unaffected with increasing environmental forcing (decrease in the mean age of 0.08%), while SBS showed higher sensitivity and decreased the mean age with increasing environmental forcing by 3.25%.

Age at maturity decreased with the intensity of size-selective fishing in both strategies and was not able to fully recover under the moratorium. The tendency towards later maturation increased with the environmental mortality rate only in single-batch spawning populations (figure 4).

## 4. Discussion

This empirically and theoretically motivated study provides a compelling perspective on how under a strong fisheries-induced selection the fitness advantage of a risk-spreading strategy can counterintuitively turn into a disadvantage. We find that reproductive strategy, in light of interactions between environmental variability and fishing mortality, can influence selection on life-history (figure 2) and fitness dynamics (figure 3). Our key finding is that while the multiple-batch spawning strategy increases the across-generational fitness under the natural selection by reducing the variance in fitness, the size-selective fisheries diminish the risk-spreading benefits of the strategy by selecting against larger sized females, that is, the phenotype that can shed the highest number of batches and thus provides the greatest risk-spreading effect. We applied several rates of natural and fishing-induced selection on simulated Atlantic cod populations with and without a multiple-batch spawning strategy. The discrete full factorial approach allowed us to distinguish the eco-evolutionary benefits of batch spawning from other life-history traits and to evaluate the extent to which this risk-spreading strategy contributes to the fitness of fished populations. Our findings support research highlighting the value that life-history diversity provides for stock productivity [36,37] (reviewed in [38]), while the individual-based modelling approach offers a novel perspective on the consequences of alternative fishing mortalities for individual fitness and population viability.

The asymptotic maximum body length during fishing rapidly decreased in our study, as we selectively started removing cod longer than 45 cm (figure 2). Given that the asymptotic length correlates with the size of maturation in our model, the fishing-induced change selected for earlier- and smaller-maturing individuals whether in the presence or absence of a multiple-batch spawning strategy. These findings are consistent with the life-history theory which predicts that size-selective fishing of larger individuals favours reduced age and size at maturation [39,40]. Life-history change in response to fishing was evident during and after fishing, having the effect of slowing subsequent population growth towards full recovery, which was never fully attained (additional tests showed no full recovery in the asymptotic length even after more than 4800 years of moratorium). The magnitude of the size difference between multiple- and single-batch spawning populations differed by 2 to 11 cm (figure 2). Given that weight (and thus fecundity) can be approximated as *L*^3^ [41], these size differences can have a substantial influence on reproductive output [29]. For example, based on weight-specific fecundity [28], a cod of 78 cm (average size of MBS before fishing) lays about 100 000 eggs fewer than a cod of 80 cm (average size of SBS before fishing). Yet, our results suggest that because of the risk-spreading effect and the distribution of eggs across a different spatial and temporal scale, ensured by the multiple-batch spawning strategy, cod do not need to grow in size as they would in the absence of the multiple-batch strategy to gain the same long-term fitness as an individual adopting the single-batch strategy. This suggests that the risk-spreading strategy could offset a smaller body size.

Our results suggest that maximizing the number of batches over the batch size is a winning reproductive tactic for cod in a highly variable environment when there is no size-selective fishing, which is similar to the benefits of maximizing the number of eggs over the egg size to ensure higher reproductive output among some fish spawners [42]. We observed that the benefit of shedding multiple batches lies in the allocation of survival probability across the environmental spectrum, which provides stability and allows the population to persist despite natural environmental perturbations. The strategy can even act as a risk-spreading strategy that safeguards fitness against environmental fluctuations [43] or a bet-hedging strategy when the uncertainty and mortality driven by the environment are high and the across-generational fitness profits from low variation in reproductive success despite the associated costs of the strategy [21,33]. Diversification of eggs reduces variance in offspring survival probability [44] and increases survival of early life stages, by increasing the probability that some offspring will experience optimal feeding conditions and experience larval development in a favourable environmental setting (e.g. hydrological and climatic).

Yet, we observed that despite the risk-spreading benefits that batch spawning provides for cod, truncation in the size structure of harvested batch spawning stocks leads to stronger losses in fitness when fishing mortality is high. These findings are consistent with studies that find the selective forces of fisheries to be very different if not the opposite of natural and have the potential to surpass natural selection in some harvested populations [45,46]. We found that the more intense the size-selective fishing, the sooner the fish matured and the lower their populational fitness was after fishing (figure 2). This outcome could be due to the evolutionary downsizing of body size. Fishing tends to truncate the size structure in harvested population, resulting in a higher proportion of adults of smaller size, producing a lower number of batches with a lower number of eggs [38,47–50].

Nonetheless, it is important to stress that our model includes several assumptions. Irrefutably these pre-set assumptions provoke a question on the possibility of variation in the model outcomes if we modify their premise. For example, some of such assumptions are the size and age at maturity, which were not under a direct fishing selection. Size at maturity was derived from its correlation to the asymptotic length while age at maturity was additionally a subject to density-dependent processes. Therefore, the possibility of a change in the direction of the evolutionary regime shift within the two traits could be challenged. Age-at-length can be a plastic trait in some fish species [51,52]. A neural network analysis of a long-term dataset showed that changes in maturation trends in stocks such as Norwegian spring-spawning herring have varied before, during and after the stock collapse period, and the reason could have been in the ecological drivers related to changing stock abundance [51]. Nevertheless, the life history of a species can affect the populational response to fishing selection. As opposed to herring, which is a pelagic SBS, demersal stocks such as cod and cod-like species, sharing a similar spawning strategy, tend to display a stronger decline in age and size at maturity [49].

The outcome of our study is consistent with empirical observations of overfished cod stocks, cautioning that fishing can be a strong selective agent, especially in populations that undergo uninterrupted heavy exploitation for several decades and can lead to a fitness deficit of heavily overfished stocks [28,46,53]. While some commercially overexploited stocks such as North Sea cod stock are showing signs of recovery [54], stocks such are Northern cod stocks struggle to recover their biomasses [13]. The results presented here warn that the abundance recovery to pre-fishing condition does not necessarily mean that the across-generational fitness has recovered as well. In other words, ecological recovery does not guarantee evolutionary recovery. For example, we found that neither multiple-batch nor their hypothetic opposite, the single-batch spawning populations, were able to recover their long-term fitness to the pre-fishing value that they had as pristine or unfished populations (figure 3). Unlike Le Bris *et al*. [55], who did not observe that batch spawning would considerably impact the population resilience of cod, our results suggest that a multiple-batch spawning strategy could additionally slow the recovery, especially after highly intense fishing (electronic supplementary material, figure S3C). The contrasting conclusion between these two modelling studies may be due to the absence of an evolutionary component in the age- and size-structured population model [55]. While the size and time needed to rebuild the biomasses are comparable, our results underscore the value of incorporating an evolving trait within mechanistic models that follow the post-fishing recovery potential. This can prove especially critical if selective fishing changes the frequency of a heritable trait (asymptotic length) that is so closely related to populational reproductive success [56].

Implications of batch spawning strategy for the management of cod stocks could be in the reduced variance of fitness. The batch spawning strategy reduces the variance in across-generational fitness, rending cod genotypes less susceptible and more resilient to environmental change, which could benefit stock predictions and return more stable catches. Our results show that if fishing mortality is low the biomass of the multiple-batch spawning population is greater, enabling fishing to last longer and consequently, return higher and more stable total catch biomass, regardless of the environmental conditions (electronic supplementary material, figures S7 and S8). The relationship between the stock spawning biomass and the recruitment is considered the beacon of fisheries science, but the metric of stock reproductive potential needs a more objective measure that embraces the nonlinearity and asymmetry of this relationship [57–59].

The diversity of reproductive strategies is indeed high [2,10,11], which makes a partition of components that define the reproductive potential highly complex, species-specific and unfeasible to generalize. We mechanistically estimated the eco-evolutionary performance of a batch spawning strategy in light of fisheries-induced evolution and found it to be yet another mechanism sensitive to highly selective human-induced mortality. Risk-spreading benefits of batch spawning strategy underpin that truncating the age and size structure of stocks such as cod truncates their ability to reduce the variance in fitness and resist natural environmental change, resulting in impaired capacity to recover.

## Data Availability

Simulation code and input files for the eco-evolutionary model used in this manuscript are available on the Dryad Digital Repository [60]. Electronic supplementary material is available online [61].
